# Genomic Characterization of *Escherichia coli* O157:H7 Associated with Multiple Sources, United States

**DOI:** 10.3201/eid3113.240686

**Published:** 2025-05

**Authors:** Joseph S. Wirth, Molly M. Leeper, Peyton A. Smith, Michael Vasser, Lee S. Katz, Eshaw Vidyaprakash, Heather A. Carleton, Jessica C. Chen

**Affiliations:** Oak Ridge Institute for Science and Education, Oak Ridge, Tennessee, USA (J.S. Wirth); Centers for Disease Control and Prevention, Atlanta, Georgia, USA (J.S. Wirth, M.M. Leeper, P.A. Smith, M. Vasser, L.S. Katz, E. Vidyaprakash, H.A. Carleton, J.C. Chen)

**Keywords:** Escherichia coli, bacteria, antimicrobial resistance, food safety, enteric infections, Shiga toxin–producing Escherichia coli, STEC, genomic sequencing, United States

## Abstract

In the United States, Shiga toxin–producing *Escherichia coli* (STEC) outbreaks cause >265,000 infections and cost $280 million annually. We investigated REPEXH01, a persistent strain of STEC O157:H7 associated with multiple sources, including romaine lettuce and recreational water, that has caused multiple outbreaks since emerging in late 2015. By comparing the genomes of 729 REPEXH01 isolates with those of 2,027 other STEC O157:H7 isolates, we identified a highly conserved, single base pair deletion in *espW* that was strongly linked to REPEXH01 membership. The biological consequence of that deletion remains unclear; further studies are needed to elucidate its role in REPEXH01. Additional analyses revealed that REPEXH01 isolates belonged to Manning clade 8; possessed the toxins *stx2a*, *stx2c*, or both; were predicted to be resistant to several antimicrobial compounds; and possessed a diverse set of plasmids. Those factors underscore the need to continue monitoring REPEXH01 and clarify aspects contributing to its emergence and persistence.

Shiga toxin–producing *Escherichia coli* (STEC) outbreaks associated with produce were first identified in 1991, and the trend of produce-associated STEC outbreaks remains prevalent, among which romaine lettuce is the most common leafy green outbreak vehicle (*1*–*4*). Each year in the United States, >265,000 STEC infections occur, costing $280 million and resulting in ≈3,600 hospitalizations and ≈30 deaths (*4*,*5*). *E. coli* O157:H7, a specific serotype of STEC, causes ≈25% of those infections and ≈67% of all STEC deaths (*5*). STEC O157:H7 infections often induce abdominal cramps, vomiting, and bloody diarrhea. In particularly severe cases, a rare condition known as hemolytic uremic syndrome (HUS) develops, which can cause anemia, acute renal failure, and death (*6*). STEC O157:H7 outbreaks are commonly linked to consumption of leafy greens or beef. Although nearly 60% of STEC O157:H7 infections have been attributed to vegetable row crops, a category that includes leafy greens, ruminants, especially cattle, are the suspected primary STEC O157:H7 reservoir (*7*–*9*). During 2009–2018, 32 STEC O157:H7 outbreaks in the United States and Canada were linked to contaminated leafy greens (*4*).

Since April 2017, nine separate outbreaks of the same strain of STEC O157:H7, hereafter referred to as REPEXH01, have occurred (Table 1). A large REPEXH01 outbreak affecting 37 states occurred in 2018, from which 238 STEC O157:H7 infections, 104 hospitalizations, 28 cases of HUS, and 5 deaths were reported (*3*). Most (85%) interviewed patients reported consuming romaine lettuce, and a subsequent investigation linked those infections to romaine lettuce grown in the Yuma, Arizona, region of the United States (*3*). By March 29, 2024, the United States reported 762 persons in 46 states infected with the REPEXH01 strain, and new infections continue to be identified. In this study, we compared whole-genome sequences of 729 REPEXH01 isolates with 2,027 other STEC O157:H7 isolates to examine genomic factors in REPEXH01 that might have contributed to the emergence and public health impacts of that strain.

**Table 1:** Outbreaks caused by reoccurring STEC O157:H7 strain REPEXH01 in a genomic characterization of *Escherichia coli* O157:H7 associated with multiple sources, United States*

Outbreak	Timeframe	Source†	Origin‡	No. reported illnesses	No. states	No. HUS cases	No. deaths	No. sequences	Allele differences(range)§
A	Apr–May 2017	Unknown	Unknown	9	5	0	0	7	3 (0–8)
B	Jul–Sep 2017	Recreational water¶	California	10	1	4	0	13	0 (0)
C	Mar–Jun 2018	Romaine lettuce¶	Arizona	238	37	28	5	238	4 (0–12)
D	Aug–Oct 2018	Ground beef#	Unknown	12	4	1	0	4	7 (4–10)
E	Oct–Dec 2018	Leafy greens#	Unknown	25	10	4	0	8	7 (1–11)
F	May–Oct 2019	Ground beef#	Unknown	44	12	4	0	44	0 (0–5)
G	Nov 2019	Unknown	Unknown	8	1	0	0	8	0 (0–1)
H	Dec 2020–Mar 2021	Unknown	Unknown	22	7	3	1	22	0 (0–0)
I	Apr–May 2021	Unknown	Unknown	5	3	0	0	5	0 (0–1)

*Outbreak dates are based on reported or estimated illness onset dates. HUS, hemolytic uremic syndrome; REPHEXHO1, recurring strain of STEC O157:H7; STEC, Shiga toxin–producing *Escherichia coli*. †Confirmed sources were implicated by epidemiologic plus traceback or laboratory data. Suspected sources were implicated by epidemiologic data only (https://www.cdc.gov/foodsafety/outbreaks/multistate-outbreaks/annual-summaries.html). ‡Geographic origin of a confirmed outbreak source might not always be known, which can happen when a food containing multiple ingredients (e.g., bagged salad blend) is confirmed as the source, but the evidence cannot implicate a specific ingredient, or when evidence confirms an outbreak source but traceback cannot pinpoint the exact geographic origin of the source. § Values indicate the median allele differences between the isolates of each respective outbreak. Values in parentheses indicate the range of minimum and maximum allele differences between the isolates of each respective outbreak. ¶Confirmed source. #Suspected source.

## Methods

### Sequence Selection and Retrieval

We used sequences from 729 REPEXH01 isolates and 598 closely related isolates previously classified as REPEXH01 for this study. All isolates were in PulseNet (https://www.cdc.gov/pulsenet/index.html) and had whole-genome sequences available in the National Center for Biotechnology Information (NCBI; https://www.ncbi.nlm.nih.gov) (Appendix 1). To compare a diverse collection of STEC O157:H7, we randomly selected 1,429 non-REPEXH01 STEC O157:H7 isolates, for a total of 2,756 genomes analyzed. That total accounts for roughly 20% of all 13,778 STEC O157:H7 isolates within PulseNet that had whole-genome sequences available in NCBI as of September 5, 2023. We downloaded whole-genome sequences from GenBank and assemblies and raw reads from the NCBI Sequence Read Archive (SRA; https://www.ncbi.nlm.nih.gov/sra) during May 23–August 1, 2023 (Appendix 2 Table 1). We used Genbank annotated genomes when available and used Prokka version 1.14.5 (*10*) to annotate SRA genomes that did not have annotations.

### Identification of Genomic Features

We used Roary version 3.11.2 (*11*) to perform pangenome analysis on Prokka-annotated genomes, then screened pangenomes for linkage to REPEXH01 isolates by using Scoary version 1.6.16 (*12*). Because those steps are computationally intensive, we used a subset of genomes comprising 181 current and 103 former REPEXH01 isolates and 2 closely related non-REPEXH01 isolates. We identified multiple alleles of *espW*, a known virulence gene, in that initial dataset and subsequently profiled the expanded dataset (n = 2,756) for those alleles and their association with REPEHX01 (*13*,*14*) (Appendix 2). We screened assemblies for antimicrobial resistance determinants, plasmid determinants, antimicrobial resistance determinant–associated point mutations, membership in O157 clades (hereafter referred to as Manning clades), and *stx* subtypes (Appendix 1).

### Phylogenetic Reconstruction

From the subset of genomes profiled for pangenome analysis, we constructed a single-nucleotide polymorphism (SNP) analysis by using Lyve-SET version 1.1.4f (https://github.com/lskatz/lyve-SET) (*15*) and presets for *Escherichia* using the single chromosomal contig of 2018C-3602 (BioSample accession no. SAMN08964444) as the reference. We used Gubbins version 3.0.0 (Sanger, https://sanger-pathogens.github.io/gubbins) to generate a recombination-free SNP alignment from the Lyve-SET core alignment (*15*,*16*). We then generated a time-scaled phylogenetic tree from the SNP alignment for a subset of 286 isolates in BEAST2 version 2.6.3 (*17*), accounting for constant sites and using bModelTest version 1.2.1 (*18*) to average across appropriate substitution models. We used BioNumerics version 7.6.3 (Applied Maths, http://www.applied-maths.com) to construct an allele-based dendrogram for 2,754 isolates by using UPGMA as the clustering technique. We excluded 2 isolates from the dendrogram because the submitting state agencies had requested those isolates be removed from PulseNet.

### Prophage Detection

We detected prophage sequences in the reference genome and categorized their genes by using the PHASTER online phage search tool (*19*,*20*). We used BLASTn version 2.14.0 (https://blast.ncbi.nlm.nih.gov) to search all *espW*-containing contigs for prophages (Appendix 1).

### Obtaining and Visualizing Isolate Metadata

Unless otherwise specified, we obtained all metadata associated with isolates in this study from the System for Enteric Disease Response, Investigation, and Coordination (SEDRIC) (https://www.cdc.gov/foodsafety/outbreaks/tools/sedric.html) or the PulseNet national database (*21*). We visualized data alongside phylogenies by using the Interactive Tree of Life version 5 webtool (https://itol.embl.de) (*22*).

## Results

### Epidemiology of REPEXH01

All REPEXH01 isolates belonged to Manning clade 8, the clade most strongly correlated with patients developing HUS (*23*,*24*). In fact, nearly every outbreak associated with the REPEXH01 strain included cases of HUS, and an average of 11% (median 9%) of reported illnesses displayed HUS (Table 1). Of the 729 REPEXH01 isolates, all possessed *stx2a*, *stx2c*, or both: 699 (96%) isolates possessed *stx2a*, 574 (79%) possessed *stx2c*, and 544 (75%) possessed both *stx2a* and *stx2c* (Appendix 2 Table 3). Because all REPEXH01 isolates belonged to Manning clade 8, those isolates likely all possessed *stx2a*, and the absence of *stx2a* in 4% of isolates was likely an artifact of the genome assemblies (*23*,*24*).

### Relationship between *espW* and REPEXH01

**Figure 1:**
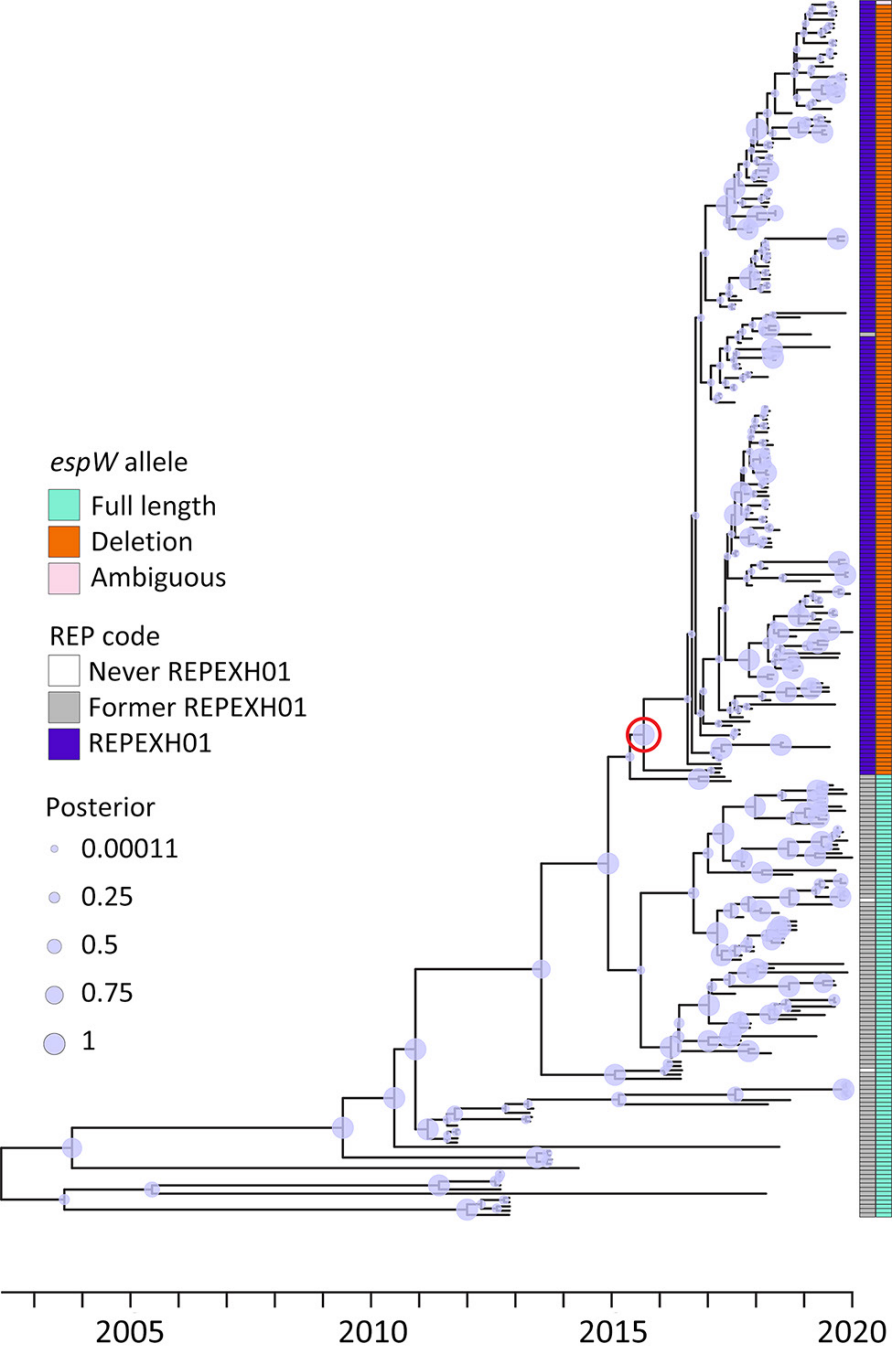
Time-calibrated tree for 286 former and current REPEXH01 isolates with associated metadata used for genomic characterization of *Escherichia coli* O157:H7 associated with multiple sources, United States. The tree was constructed using BEAST2 (https://beast.community) on an alignment of high-quality single-nucleotide polymorphisms. The red circle indicates the most common recent ancestor of the REPEXH01 isolates and corresponds to December 2015. On the right side, the first column indicates the current REPEXH01 isolates (purple), former REPEXH01 isolates (gray), or isolates that were never part of the REPEXH01 definition (white). The second column indicates the *espW* allele: teal indicates the full-length allele, orange indicates the presence of a single base pair deletion; and pink indicates that *espW* is present but the allele could not be determined due to inadequate sequencing data. Circles on the branches indicate the posterior probability. REP, reocurring, emerging, and persistent; REPHEXH01, recurring strain of Shiga toxin–producing *E. coli* O157:H7.

**Figure 2:**
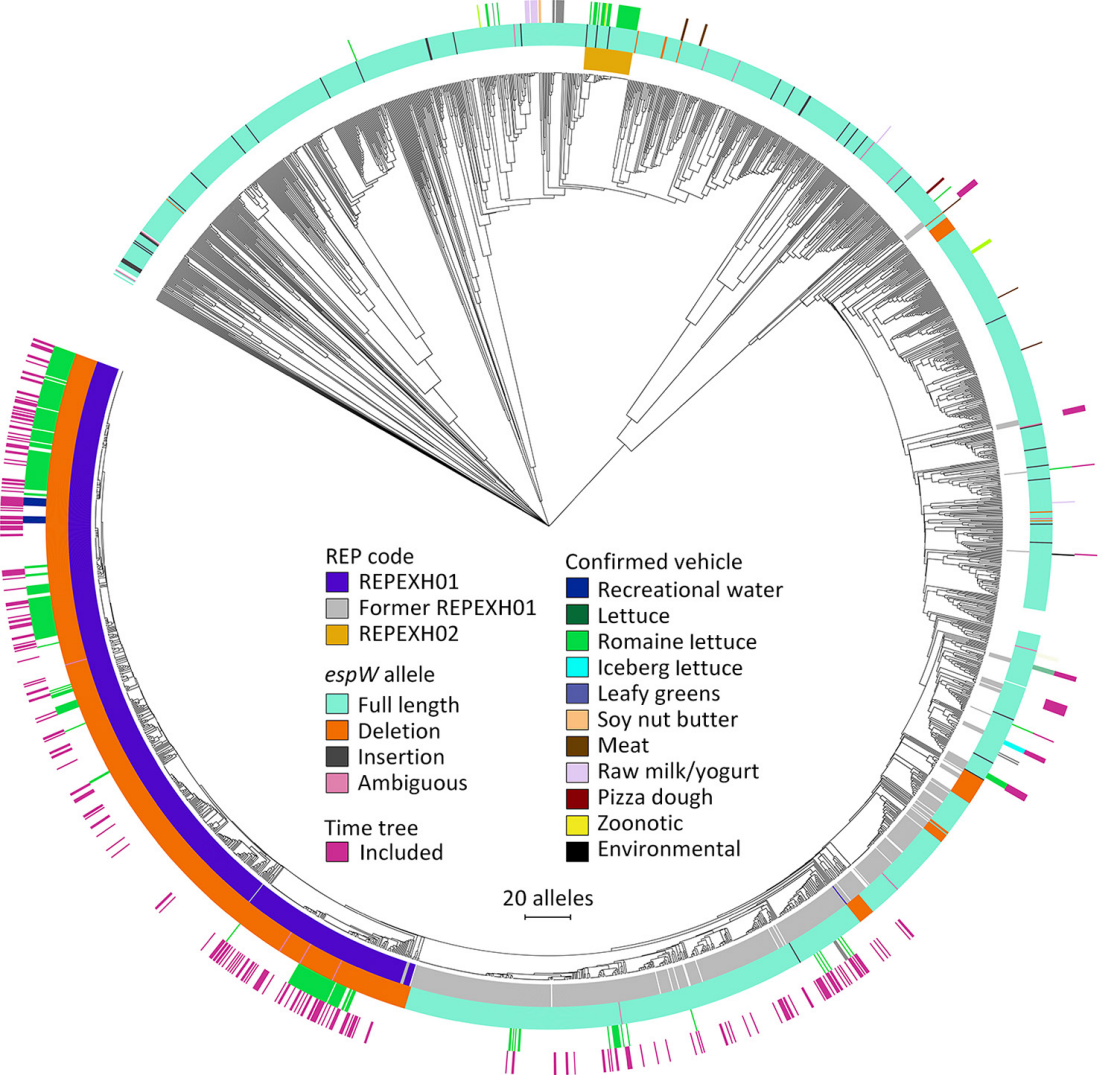
Allele-based core genome multilocus sequence typing dendrogram for 2,752 STEC O157:H7 isolates and associated metadata used for genomic characterization of *Escherichia coli* O157:H7 associated with multiple sources, United States. The dendrogram was constructed in BioNumerics version 7.6.3 (Applied Maths, http://www.applied-maths.com) by using UPGMA as the clustering technique. The innermost track indicates whether an isolate was included in a STEC O157:H7 REP strain. The second innermost track indicates the *espW* allele showing the full-length allele, single base pair deletions or insertion, where *espW* allele could not be determined due to inadequate sequencing data, and where *espW* was not detected in the isolate. The second outermost track indicates vehicles that have been confirmed via epidemiologic investigations of coded outbreaks. The outermost track indicates whether an isolate was included in the time-calibrated tree (Figure 1). An interactive version of this tree is available at https://itol.embl.de/tree/1581112362321361691692003. The following isolates were omitted from this tree: National Center for Biotechnology Information Sequence Read Archive (https://www.ncbi.nlm.nih.gov/sra) accession nos. SRR7094189, SRR8956189, SRR7540755, and SRR5588761. The scale bar indicates the number of allele differences between isolates. REP, reocurring, emerging, and persistent; REPHEX01, recurring strain of STEC HO1:H7; STEC, Shiga toxin–producing *Escherichia coli*.

We performed a preliminary Roary/Scoary pangenome analysis on a subset of 264 isolates, which indicated that the presence of *espW* was linked to membership in REPEXH01, but that same linkage was absent when analyzing the 286 isolates in the time-scaled tree (Figure 1). Closer inspection revealed that *espW* was in all isolates but often possessed a conserved single base pair deletion, and that deletion appeared to be linked to REPEXH01. We confirmed that hypothesis by analyzing the *espW* alleles in 2,756 isolates, 729 of which were REPEXH01, 598 were former REPEXH01 isolates, and the other 1,429 were a random sampling of all other STEC O157:H7 isolates in the PulseNet database that had publicly available genomes in NCBI (Table 2, Figure 2; Appendix 2 Table 2). We used a χ^2^ statistical test, ignoring ambiguous data, to examine the relationship between *espW* alleles and REPEXH01 membership and found the association between those variables was significant (p<0.0001). REPEXH01 isolates were more likely to have the deletion than other STEC O157:H7 isolates.

**Table 2:** Distribution of *espW* alleles in the 2,756 STEC O157:H7 isolates included in a genomic characterization of *Escherichia coli* O157:H7 associated with multiple sources, United States*

REP code	*espW* allele	Count
REPEXH01	Full length	0
	Deletion	727
	Insertion	0
	Ambiguous	2
	Absent	0
non-REPEXH01	Full length	1,892
	Deletion	77
	Insertion	22
	Ambiguous	6
	Absent	30

*Values represent 20% of all STEC O157:H7 isolates in the PulseNet (https://www.cdc.gov/pulsenet/index.html) database with whole-genome sequences available in the National Center for Biotechnology Information Sequence Read Archive (https://www.ncbi.nlm.nih.gov/sra); all 729 REPEXH01 isolates with whole-genome sequences are included, alongside 2,027 non-REPEXH01 STEC O157:H7 isolates. The *espW* allele was statistically associated with REPEXH01 membership. REPHEXH01, recurring strain of STEC O157:H7; STEC, Shiga toxin–producing *Escherichia coli*.

The deletion in *espW* consisted of the loss of a single adenine residue, converting a homopolymer within codons 174–176 from 8 adenine to 7 adenine residues. That deletion introduced a frameshift that ultimately resulted in an early termination codon. We observed insertion of an adenine residue, from 8 to 9 residues, within the same locus in 22 isolates. That insertion also introduced an early termination codon.

### REPEXH01 Emergence

Analysis of 286 current and former REPEXH01 isolates revealed that the strain emerged around December 23, 2015 (95% highest posterior density interval March 5, 2015–September 4, 2016), before it was detected in clinical cases in April 2017 (Figure 1). That phylogeny appeared to suggest that members of REPEXH01 shared a common ancestor and the single base pair deletion in *espW* associated with REPEXH01 appeared to coincide with the emergence of the REPEXH01 strain in late 2015 (Figure 1).

### *espW* Association with STEC O157:H7 Prophages

**Figure 3:**
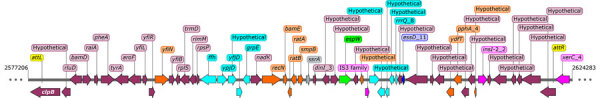
Prophage architecture for most *espW*-containing isolates from a genomic characterization of *Escherichia coli* O157:H7 associated with multiple sources, United States. This locus was annotated using PROKKA version 1.14.5 (https://github.com/tseemann/prokka) and represents a 47,078-bp sequence of Shiga toxin–producing *E. coli* O157:H7 PNUSAE013304 (BioSample accession no. SAMN0896444; SRA accession no. SRR7050023) from positions 2577206–2624283. Arrows indicate genes and their direction indicates the DNA strand. *att* sites were annotated by PHASTER (https://phaster.ca) and are shown in yellow. Genes are colored on the basis of the category assigned by PHASTER: teal indicates phage tail; orange indicates phage-like proteins; pink indicates transposases, integrases, or recombinases; blue indicates phage lysis proteins; gray indicates tRNA; and maroon indicates genes that were not categorized by PHASTER. *espW* is highlighted in green. IS, insertion.

Examining the gene synteny surrounding *espW* in the reference sequence for REPEXH01 (BioSample accession no. SAMN0896444) showed that many neighboring genes appeared to be of phage origin. Analyzing that genome using PHASTER (*20*) revealed that *espW* was contained within a putative prophage that was most closely related to *Escherichia* phage 500465-1 (GenBank accession no. NC_049342.1) (Figure 3). We examined the genomic regions containing *espW*, and most isolates possessed *espW* within the same putative prophage (Appendix 2 Table 2). Although we detected additional loci in ≈43 isolates, most were of phage origin. Of the 2,626 isolates with assembled contigs that contained *espW*, 87% (n = 2,292) possessed *espW* in or near a putative prophage region (Appendix 2 Table 2). One isolate (SRA accession no. SRR93211959) possessed *espW* directly adjacent to a prophage in what appeared to be an effector exchange locus (*13*). Another isolate (SRA accession no. SRR6870099) contained *espW* in a nonprophage region. In the other 332 (13%) isolates, presence of *espW* in a phage-associated region was ambiguous.

### Additional REPEXH01 Genomic Features 

We evaluated antimicrobial resistance determinants in REPEXH01 (Table 3; Appendix 2 Table 3). REPEXH01 is known to be resistant to several antimicrobial drugs and our dataset confirmed that resistance (*25*). Of note, our results predicted that >99% of REPEXH01 isolates would be resistant to aminoglycosides, folate pathway inhibitors, phenicols, quaternary ammonium compounds, sulfonamides, and tetracylines. However, data predict few isolates would be resistant to cephalosporins (<2%), fluoroquinolones (<1%), or penicillins (<1%).

**Table 3:** Antimicrobial resistance determinants in the 729 REPEXH01 isolates in a genomic characterization of *Escherichia coli* O157:H7 associated with multiple sources, United States*

Antimicrobial class	% Resistant isolates
Aminoglycosides†	99.6
Folate pathway inhibitors‡	99.6
Phenicols§	99.6
Sulfonamides¶	99.6
Quaternary ammonium compounds#	99.6
Tetracyclines**	99.5
Cephalosporins††	1.9
Fluoroquinolones‡‡	0.3
Penicillins§§	0.3

*Antimicrobial resistance determinants were determined by using ResFinder (https://cge.cbs.dtu.dk/services/ResFinder). Resistance was defined by the presence of one or more determinants. REPHEXH01, recurring strain of Shiga toxin–producing *Escherichia coli* O157:H7. †*aadA1*, *aph(3”)-Ib*, and *aph (**6**)-Id*. ‡*dfrA1* and *dfrA8.*§*floR*. ¶*sul1* and *sul2*. #*qacE*. **tet(A) and *tet(B)*. ††*bla*CMY-2 and *bla*_CTX-M-27_. ‡‡*qnrB19*. §§*bla*_TEM-1B_.

We also investigated REPEXH01 plasmids (Table 4; Appendix 2 Table 3) and detected >1 plasmid replicons in >95% of isolates. Most isolates possessed the IncFIB replicon, IncFIA replicon, or both replicons, but other replicons were not as prevalent. Approximately 9% of isolates contained IncFII replicons, but IncI1-Iγ, IncI2, IncB/O/K/Z, Col, IncX4, and pEC4115 were detected in <5% of isolates.

**Table 4:** Plasmid content in the 729 REPEXH01 isolates from a genomic characterization of *Escherichia coli* O157:H7 associated with multiple sources, United States*

Plasmid replicon	% Isolates
IncFIB	92.7
IncFIA	92.3
IncFII†	8.8
IncI1-I(gamma)	4.9
IncI2‡	3.6
IncB/O/K/Z	1.5
Col§	0.8
IncX4	0.7
pEC4115	0.7
IncFIC(FII)	0.3

*Presence of plasmid replicons was determined by using PlasmidFinder (Center for Genomic Epidemiology, https://genomicepidemiology.org). REPHEXH01, recurring strain of Shiga toxin–producing *Escherichia coli* O157:H7. †IncFII, IncFII(29), IncFII(pCoo), IncFII(pHN7A8), and IncFII(pSE11). ‡IncI2 and IncI2(delta). §ColE1, ColpVC, Col(KPHS6), Col(MG828), and Col(pHAD28).

## Discussion

A key finding in this study was identification of a SNP mutation in the *espW* gene that is largely characteristic of the REPEXH01 strain. The EspW protein has been shown to be secreted by a type III secretion system (T3SS) in *E. coli* O157:H7 and was previously observed within effector exchange locus (*13*). Once secreted into the host intestinal epithelial cell, EspW reorganizes host-cell actin in a Rac1-dependent manner to enable extracellular attachment (*14*). A *Pseudomonas syringae* homolog of that protein, HopW1, has been shown to solubilize cytosolic actin when injected into plant cells by a T3SS, which disrupts normal localization of proteins and might interfere with the plant immune response (*26*). The T3SS and secretory proteins such as EspA have been shown to play integral roles in the colonization of the surface of leaves and deeper tissues of the phyllosphere in spinach and lettuce, where STEC O157:H7 can continue to grow under favorable conditions (*27*,*28*). 

However, the biological significance of the single base pair deletion in *espW* remains unclear. That deletion could be an example of a gene truncation; another study observed truncations of *espW* in other pathogenic strains of *E. coli* (*14*). Alternatively, the resulting frame shift might silence expression of *espW*, or *espW* might be regulated by a homopolymeric tract mechanism where slippage of RNA polymerase could produce heterogenous transcripts, some of which could encode the in-frame functional gene product (*29*). In each of those scenarios, reduced EspW could promote colonization of romaine lettuce through several mechanisms. For example, EspW might elicit an immune response from an infected plant, causing stomata to close, thus restricting access to the interior of leaves by colonizing STEC. Alternatively, EspW could function like HopW1 and cause a more severe infection in plant tissues, lowering the likelihood that the infected leaves are harvested and consumed. Further experiments are required to elucidate the role of that single base pair deletion in REPEXH01 isolates.

In this study, we performed key molecular profiling to provide information on molecular attributes of REPEXH01. Certain *stx* subtypes are associated with more severe disease, and the prevalence of *stx2a* in REPEXH01 highlights the need for surveillance of this strain (*30*). All isolates of this strain belonged to Manning clade 8, the clade most strongly correlated with poor disease outcomes (*23*,*24*). Nearly all REPEXH01 isolates possessed antimicrobial resistance determinants, but that finding does not have direct clinical significance because antimicrobial drugs are not indicated for treating STEC infections because those drugs can increase toxin concentrations in the patient (*25*). However, the plasmids observed in REPEXH01 isolates have been implicated in horizontal gene transfer, and those plasmids were in >95% of REPEXH01 isolates (Appendix 2 Table 3) (*31*). Taken together, those findings suggests that although the presence of antimicrobial resistance determinants has minimal effects on clinical outcomes of STEC infections, and REPEXH01 isolates could still serve as a reservoir of antimicrobial resistance.

Among the limitations of this study, although we included all current and former REPEXH01 isolates in this study, we only screened 20% of the total STEC O157:H7 isolates to decrease the computational demand of the analyses. That subsampling has the potential to bias the data, but the random selection of non-REPEXH01 STEC O157:H7 genomes might alleviate that bias. The genomes used in this study were primarily derived from short-read sequencing technology, and most were at the draft level, indicating that the replicons had not been fully assembled. Although use of draft genomes could result in *espW* being erroneously called absent, steps such as read recruitment using ARIBA (https://github.com/sanger-pathogens/ariba) helped mitigate those potential errors.

REPEXHO1 is a persistent strain of STEC O157:H7 that we estimate emerged in late 2015, before the detection of clinical cases beginning in April 2017. We detected a single base pair deletion in the *espW* virulence gene in >99% of REPEXH01 isolates but in only a few (<4%) non-REPEXH01 STEC O157:H7 isolates (Table 2). That deletion can be useful as a genomic signature of this strain for molecular surveillance and as a subject of future research to clarify the strain’s evolution. Additional research addressing the role of the single base pair mutation in this strain’s colonization and survival on leafy vegetables could yield valuable insights. 

In summary, REPEHX01 belongs to *E. coli* O157:H7 Manning Clade 8, and most isolates possess *stx2a*, both factors that are associated with severe clinical outcomes. Those factors, along with its harboring of multiple resistance determinants, underscore the continued need to monitor REPEXH01 and understand factors contributing to its emergence and persistence.

Appendix 1 Additional information on genomic characterization of *Escherichia coli* O157:H7 Associated with multiple sources, United States.

Appendix 2 Genomic information for characterization of *Escherichia coli* O157:H7 Associated with multiple sources, United States.
