# Supplementary material for: Genomic Characterization of Escherichia coli O157:H7 Associated with Multiple Sources, United States
Source: Emerg Infect Dis. 2025 May;31(Suppl 1):S109–16. doi: 10.3201/eid3113.240686 (PMC12078548; doi:10.3201/eid3113.240686)
Supplement: Appendix 1 — Additional information on genomic characterization of Escherichia coli O157:H7 Associated with multiple sources, United States. [file 24-0686-Techapp-s1.pdf]

Article DOI: https://doi.org/10.3201/eid3113.240686

EID cannot ensure accessibility for supplementary materials supplied by authors. Readers who have difficulty accessing supplementary content should contact the authors for assistance.

Genomic Characterization of *Escherichia coli* O157:H7 Associated with Multiple Sources, United States

Appendix 1

Supplemental Methods

Sequence Selection and Retrieval

All sequence data used in this study met minimum standards for submission to the PulseNet National database. To be submitted to PulseNet a sequence must be identified as the target genus by ANI, be free of contamination, have an average genome coverage of at least 40x (for *Escherichia*), and an average score greater than or equal to 30. Assembled genomes must fall between 4.9–5.9 MB in length.

REPEXH01 Strain Definition

In REPEXH01 isolates were defined as being related within 0–27 alleles by core genome multilocus sequence typing (cgMLST) and with an allele code designation of EC1.0 - 9.1.3.70x. Allele codes were developed and used by PulseNet, the national molecular subtyping network for foodborne disease surveillance in the United States. Allele codes provide a hierarchical name to show relatedness between isolates based on cgMLST and are used within PulseNet to help identify disease clusters or as a form of nomenclature for referencing strains (*1*,*2*). As of July 5, 2023, 730 isolates in PulseNet met this definition. All 598 closely related isolates previously classified as REPEXH01 but subsequently reclassified were also included. These isolates were removed from the strain definition of REPEXH01 to narrow this strain to focus on a group of more closely related isolates primarily associated with past outbreaks linked to leafy greens and recreational water.

Screening *espW* Alleles

The multiple alleles of *espW* (i.e., deletion, full length, insertion) were used as a query against a database of all isolate contigs using BLASTn v 2.14.0 with the following parameters: 90% identity, 35% query coverage, ungapped alignments, and 10,000 maximum target sequences (*3*). For isolates where BLASTn did not identify *espW* in the assembled genome sequences, ARIBA v 2.12.0 was used to assemble to the *espW* gene from recruited reads using the default settings, and these assembled contigs were subsequently used to identify the *espW* allele (*4*). This strategy helped reduce false negatives due to assemblies with low contiguity. This workflow has been packages as a command line tool and is available on GitHub (https://github.com/ncezidbiome/espwAlleleCaller).

Identification of Genomic Features

Assemblies were screened for antimicrobial resistance determinants (ARDs) and were identified using staramr v 0.7.2 and the ResFinder database (last updated February 4, 2022) (*5*,*6*). Plasmid determinants were screened using abricate v 1.0.1 and a custom version of the PlasmidFinder database (https://github.com/StaPH-B/resistanceDetectionCDC/blob/master/plasmidDatabase.fasta) (*7*,*8*). Point mutations associated with resistance were screened from raw reads using ARIBA v 2.12.0 and the PointFinder database (last updated July 2, 2019) (*4*,*9*). To provide important risk context, four informative SNPs (ECs2357, Ecs2521, Ecs3881, and Ecs4130) were used to determine membership in O157 clades (hereafter referred to as Manning clades) initially described by Manning *et al*., which have been shown to have variable associations with severe disease, namely HUS (*10*,*11*). Using previously reported primers (*12*), PCR was performed in silico (https://github.com/ucscGenomeBrowser/kent/tree/master/src/isPcr) to determine the *stx* gene subtypes as previously described (*11*).

Phylogenetic Reconstruction

SNP analyses and molecular clock analyses is computationally intensive, therefore we aimed to keep our subsample under 300 isolates. We aimed to include early sequences to help root the tree, and included all available sequences that met the criteria of the REP strain or were identified to be closely related that were isolated through 2017, and sampled up to 100 sequences from the years 2018 and 2019 and included available sequences from 2020. Ultimately this REP

strain was reclassified as described previously and led to the designation of "former REPEXH01" isolates in this dataset.

Single nucleotide polymorphism (SNP) analysis was performed on each subset was using LyveSET v 1.1.4f with the presets for *Escherichia* using the single chromosomal contig of 2018C-3602 (NCBI accession: SAMN08964444) as the reference (*13*). Reads were cleaned using CG pipeline as employed in Lyve-SET. Gubbins v. 3.0.0 was used to generate a recombination free alignment (*14*). Isolates with more than 25% missing data were removed from the alignment. Output was analyzed using TempEST to assess temporal signal and identify outliers which were subsequently removed from the alignment (*15*,*16*).

Exponential and Bayesian skyline tree priors were evaluated using both strict clock and lognormal relaxed clock methods in BEAST2 v 2.6.3, using bModel test v. 1.2.1 to perform substitution model averaging (*15*,*17*). The XML file from beauti was modified using the numbers of constant sites obtained by interrogating the pooled vcf file output from Lyve-SET. MCMC chains were run for 500,000,000 iterations with sampling every 50,000 iterations. Log files were inspected using Tracer v. 1.6. Chosen models converged and had strong ESS values in Tracer. Ultimately, we selected the Bayesian skyline model using the relaxed clock, since there was a sizable coefficient of variation around the clock rate observed in the relaxed clock indicating variability across branches, and a skyline model as it's best suited to analyzed data where little is known about the population dynamics. TreeAnnotator was used to generate a maximum clade credibility tree using the "keep" option for height, setting the burnin at 10% and the posterior cutoff at 0.9.

Prophage Identification in *espW*-containing Contigs

BLASTn v 2.14.0 was used to search all *espW*-containing contigs for prophages (see Supplemental Material for more information). as queries against a putative prophage with the following parameters: 1 maximum high-scoring pair and 10,000 maximum target sequences. If ≥90% of the prophage was covered by the BLAST hit or if ≥50% of the prophage was covered and ≥90% of the contig was covered by the BLAST hit, then the *espW* locus was classified as phage associated. If <50% of the phage was covered and ≥90% of the contig was covered, then the *espW* locus was classified as ambiguous. If <90% of the phage was covered and <90% of the contig was covered, then the *espW* locus was manually investigated.

References

1. Stevens EL, Carleton HA, Beal J, Tillman GE, Lindsey RL, Lauer AC, et al. Use of whole genome sequencing by the Federal Interagency Collaboration for Genomics for Food and Feed Safety in the United States. J Food Prot. 2022;85:755–72. PubMed https://doi.org/10.4315/JFP-21-437

2. Zhou Z, Alikhan N-F, Mohamed K, Fan Y, Achtman M; Agama Study Group. The EnteroBase user's guide, with case studies on *Salmonella* transmissions, *Yersinia pestis* phylogeny, and *Escherichia* core genomic diversity. Genome Res. 2020;30:138–52. PubMed https://doi.org/10.1101/gr.251678.119

3. Camacho C, Coulouris G, Avagyan V, Ma N, Papadopoulos J, Bealer K, et al. BLAST+: architecture and applications. BMC Bioinformatics. 2009;10:421. PubMed https://doi.org/10.1186/1471-2105-10-421

4. Hunt M, Mather AE, Sánchez-Busó L, Page AJ, Parkhill J, Keane JA, et al. ARIBA: rapid antimicrobial resistance genotyping directly from sequencing reads. Microb Genom. 2017;3:e000131. PubMed https://doi.org/10.1099/mgen.0.000131

5. Bharat A, Petkau A, Avery BP, Chen JC, Folster JP, Carson CA, et al. Correlation between phenotypic and *in silico* detection of antimicrobial resistance in *Salmonella enterica* in Canada using staramr. Microorganisms. 2022;10:292. PubMed https://doi.org/10.3390/microorganisms10020292

6. Florensa AF, Kaas RS, Clausen PTLC, Aytan-Aktug D, Aarestrup FM. ResFinder - an open online resource for identification of antimicrobial resistance genes in next-generation sequencing data and prediction of phenotypes from genotypes. Microb Genom. 2022;8:000748. PubMed https://doi.org/10.1099/mgen.0.000748

7. Seemann T. abricate. 2024 [cited 2024 Feb 9]. https://github.com/tseemann/abricate

8. Carattoli A, Zankari E, García-Fernández A, Voldby Larsen M, Lund O, Villa L, et al. *In silico* detection and typing of plasmids using PlasmidFinder and plasmid multilocus sequence typing. Antimicrob Agents Chemother. 2014;58:3895–903. PubMed https://doi.org/10.1128/AAC.02412-14

9. Zankari E, Allesøe R, Joensen KG, Cavaco LM, Lund O, Aarestrup FM. PointFinder: a novel web tool for WGS-based detection of antimicrobial resistance associated with chromosomal point mutations in bacterial pathogens. J Antimicrob Chemother. 2017;72:2764–8. PubMed https://doi.org/10.1093/jac/dkx217

10. Riordan JT, Viswanath SB, Manning SD, Whittam TS. Genetic differentiation of *Escherichia coli* O157:H7 clades associated with human disease by real-time PCR. J Clin Microbiol. 2008;46:2070–3. PubMed https://doi.org/10.1128/JCM.00203-08

11. Chen JC, Patel K, Smith PA, Vidyaprakash E, Snyder C, Tagg KA, et al. Reocurring *Escherichia coli* O157:H7 strain linked to leafy greens–associated outbreaks, 2016–2019. Emerg Infect Dis. 2023;29:1895–9. PubMed https://doi.org/10.3201/eid2909.230069

12. Scheutz F, Teel LD, Beutin L, Piérard D, Buvens G, Karch H, et al. Multicenter evaluation of a sequence-based protocol for subtyping Shiga toxins and standardizing Stx nomenclature. J Clin Microbiol. 2012;50:2951–63. PubMed https://doi.org/10.1128/JCM.00860-12

13. Katz LS, Griswold T, Williams-Newkirk AJ, Wagner D, Petkau A, Sieffert C, et al. A comparative analysis of the lyve-SET phylogenomics pipeline for genomic epidemiology of foodborne pathogens. Front Microbiol. 2017;8:375. PubMed https://doi.org/10.3389/fmicb.2017.00375

14. Croucher NJ, Page AJ, Connor TR, Delaney AJ, Keane JA, Bentley SD, et al. Rapid phylogenetic analysis of large samples of recombinant bacterial whole genome sequences using Gubbins. Nucleic Acids Res. 2015;43:e15. PubMed https://doi.org/10.1093/nar/gku1196

15. Bouckaert R, Heled J, Kühnert D, Vaughan T, Wu C-H, Xie D, et al. BEAST 2: a software platform for Bayesian evolutionary analysis. PLOS Comput Biol. 2014;10:e1003537. PubMed https://doi.org/10.1371/journal.pcbi.1003537

16. Rambaut A, Lam TT, Max Carvalho L, Pybus OG. Exploring the temporal structure of heterochronous sequences using TempEst (formerly Path-O-Gen). Virus Evol. 2016;2:vew007. PubMed https://doi.org/10.1093/ve/vew007

17. Bouckaert RR, Drummond AJ. bModelTest: Bayesian phylogenetic site model averaging and model comparison. BMC Evol Biol. 2017;17:42. PubMed https://doi.org/10.1186/s12862-017-0890-6
